# Supplementary material for: Beat frequency quartz-enhanced photoacoustic spectroscopy for fast and calibration-free continuous trace-gas monitoring
Source: Nat Commun. 2017 May 31;8:15331. doi: 10.1038/ncomms15331 (PMC5460019; doi:10.1038/ncomms15331)
Supplement: Supplementary Information — Supplementary Figures, Supplementary Notes and Supplementary References. [file ncomms15331-s1.pdf]

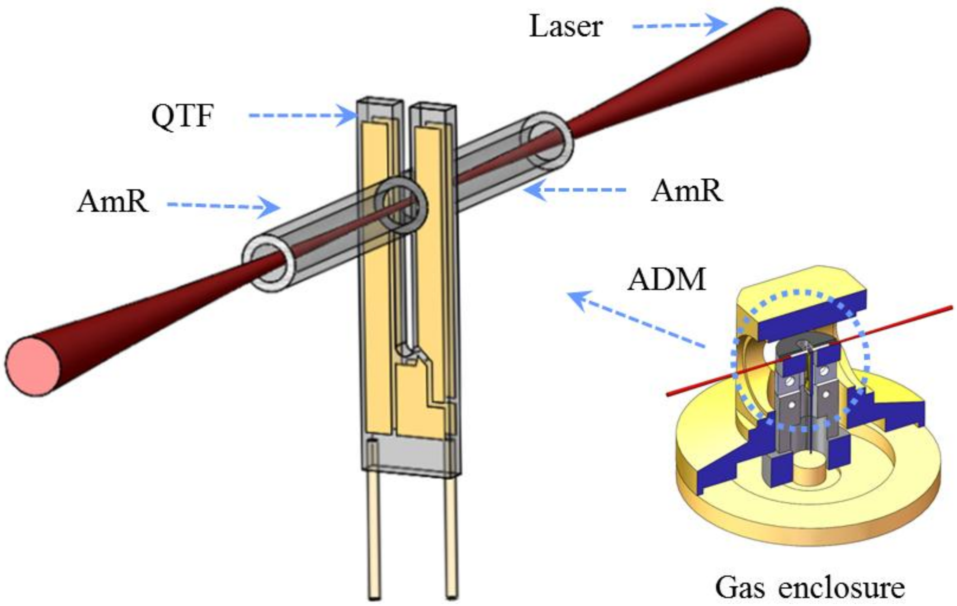

Supplementary Figure 1: Enlarged image for an on-beam configuration acoustic detection module. The acoustic detection module (ADM) consists of a standard commercial QTF and a set of acoustic micro-resonators (AmRs)[1]. A commercially available millimeter sized QTF was used as the acoustic transducer due to the piezoelectric effect of the quartz crystal. This standard QTF has resonance frequency of 32.7 kHz with prong lengths of 3.8 mm, prong widths of 0.6 mm and gap between QTF's prongs of 0.3 mm. The AmRs were placed on the two sides of the QTF to enhance the photoacoustic spectroscopy (PAS) signal. The laser radiation must pass through the AmRs and the gap between QTF's prongs without touching them, as otherwise the laser radiation blocked by AmRs or QTF results in a non-zero background.

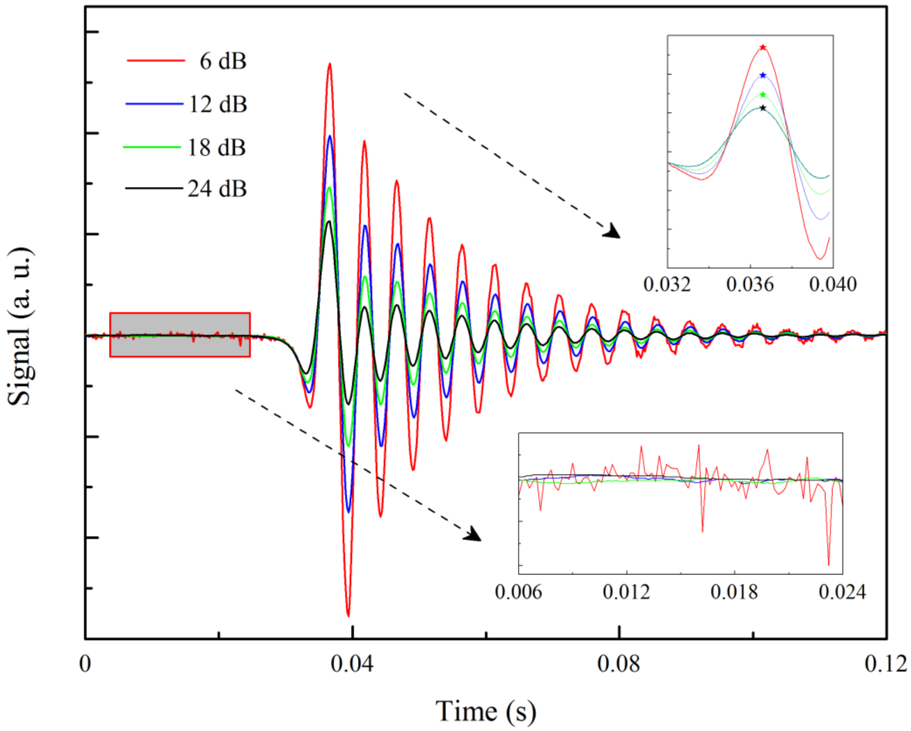

Supplementary Figure 2: BF-QEPAS signal for different filter slopes of the LIA. The DFB diode laser emitting at 1.368 μm was used as the excitation source. The modulation frequency and depth of the wavelength was 32,960 Hz and 1 cm^{-1} (29 mA), respectively. The LIA time constant was set at 100 μs. The wavelength was scanned at the rate of 36 cm^{-1} s^{-1}. The signals were detected at room temperature and 760 Torr when the ADM was filled with 2.5 % water vapor.

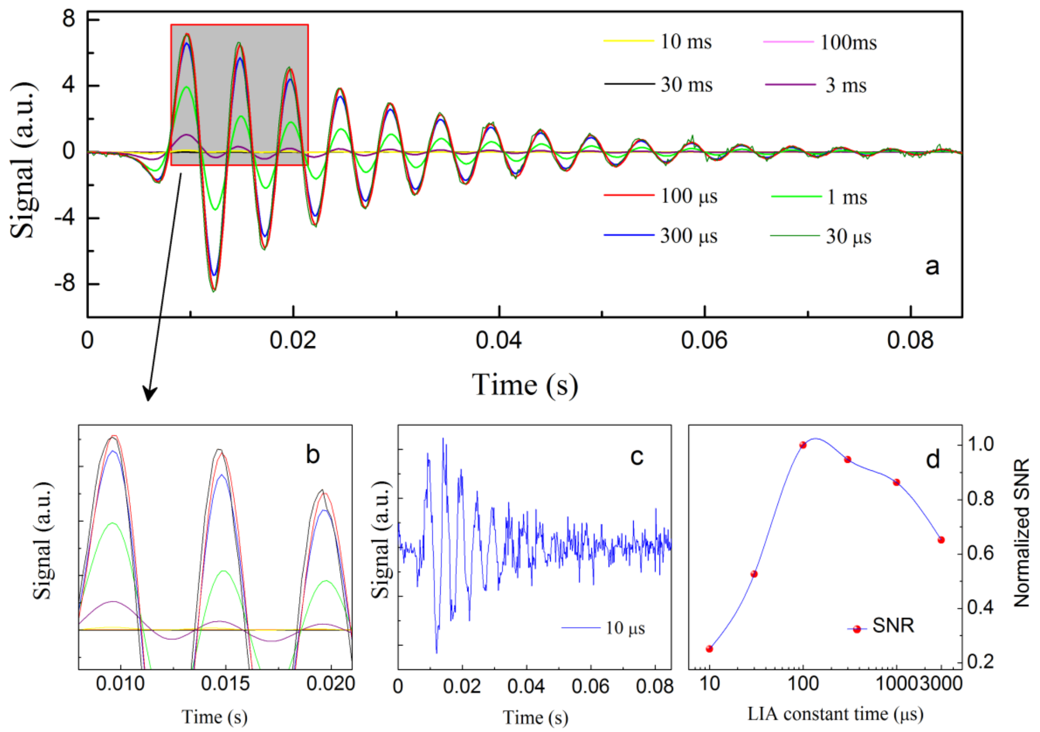

Supplementary Figure 3: BF-QEPAS signal for different LIA time constant. (a)-(c) BF-QEPAS signal for different LIA time constants. (d) Normalized SNR value for different LIA time constants. A DFB diode laser emitting at 1.368 µm was used as the excitation source. The modulation frequency and depth of the wavelength was 32,960 Hz and 1 cm^{-1}, respectively. The filter slope of the LIA was set at 12 dB. The wavelength was scanned at the rate of 36 cm^{-1} s^{-1}. The signals were detected at room temperature and 760 Torr, when the ADM was filled with 2.5 % water vapor. The SNR value corresponding to the 10 ms, 30 ms and 100 ms were not calculated and plotted in (d), as the LIA detection bandwidth was too narrow to detect the perfect BF-QEPAS signal when the LIA time constant was >3 ms.

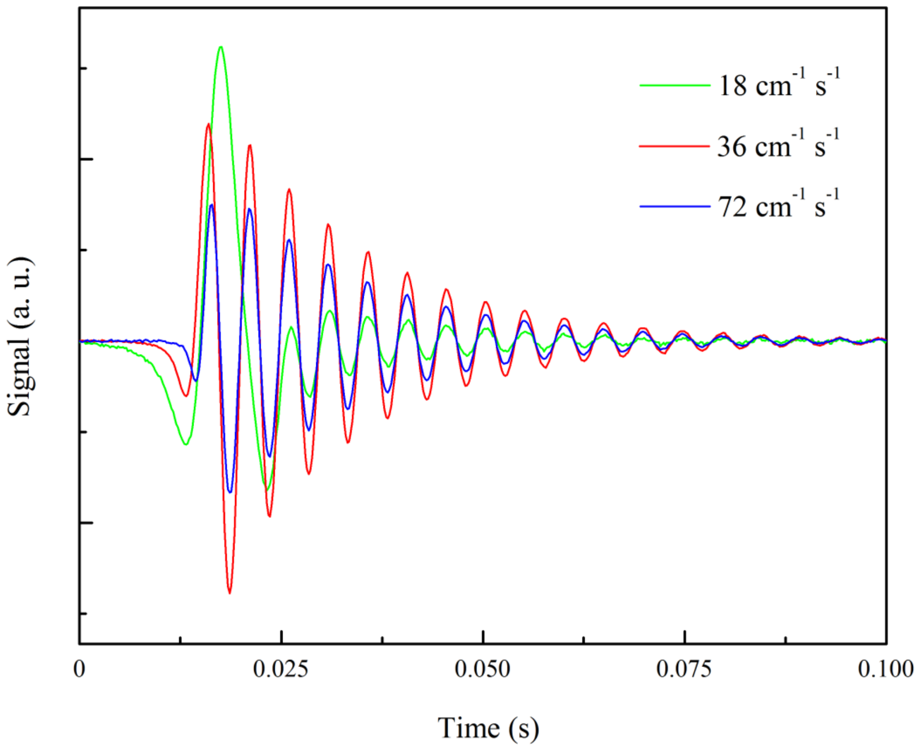

Supplementary Figure 4: BF-QEPAS signal for different wavelength scanning rates. The DFB diode laser was used as the excitation source. The modulation frequency and depth of the wavelength was 32,960 Hz and 1 cm^{-1}, respectively. The time constant and filter slope of the LIA was set at 100 μs and 12 dB, respectively. The signals were detected at room temperature and 760 Torr when the ADM was filled with 2.5 % water vapor.

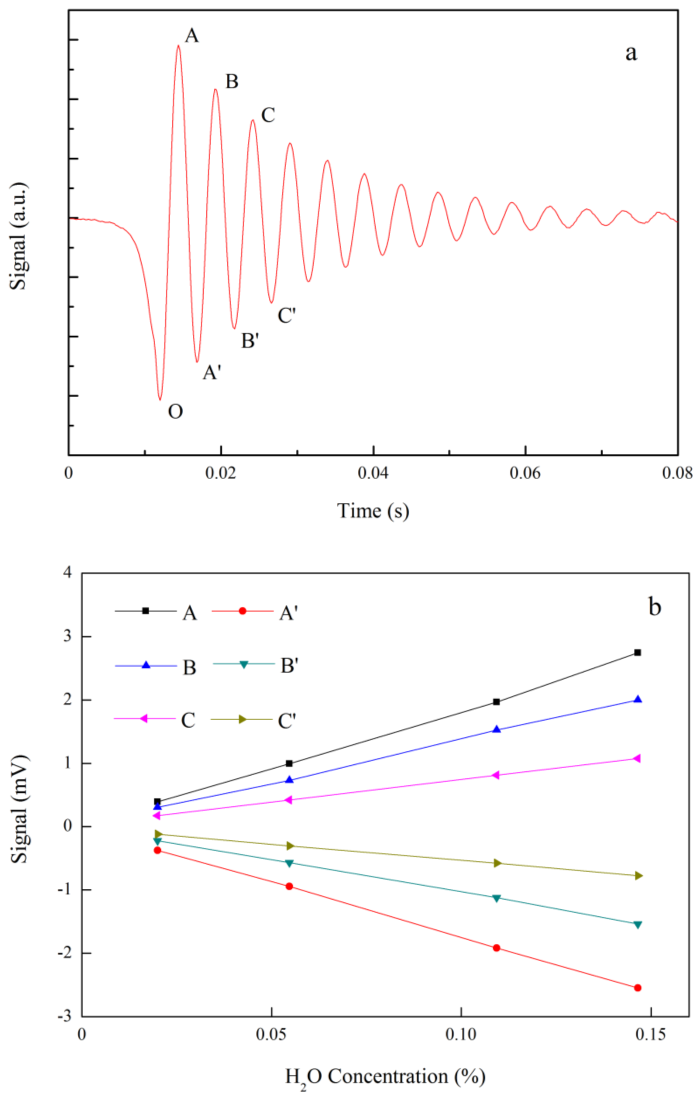

Supplementary Figure 5: 1*f* based BF-QEPAS signal and linear dependence of peaks/valleys of the BF-QEPAS signal for different H_2O concentrations. A DFB diode laser emitting at 1.368 μm was used as the excitation source. The modulation frequency and depth of the wavelength was 32,960 Hz and 1 cm^{-1} (29 mA), respectively. The wavelength-scanning rate of the diode laser, time constant and filter slope of the Lock-in amplifier (LIA) for BF-QEPAS technique were 36 cm^{-1} s^{-1}, 100 μs and 12 dB, respectively. In (a), the first three peaks of the BF-QEPAS signal were marked as A, B and C. Similarly, the first four valleys of the signal were marked as O, A', B' and C'. In (b), the amplitudes of the peaks A-C and valleys A'-C', acquired with different H_2O concentrations were plotted as a function of H_2O concentrations.

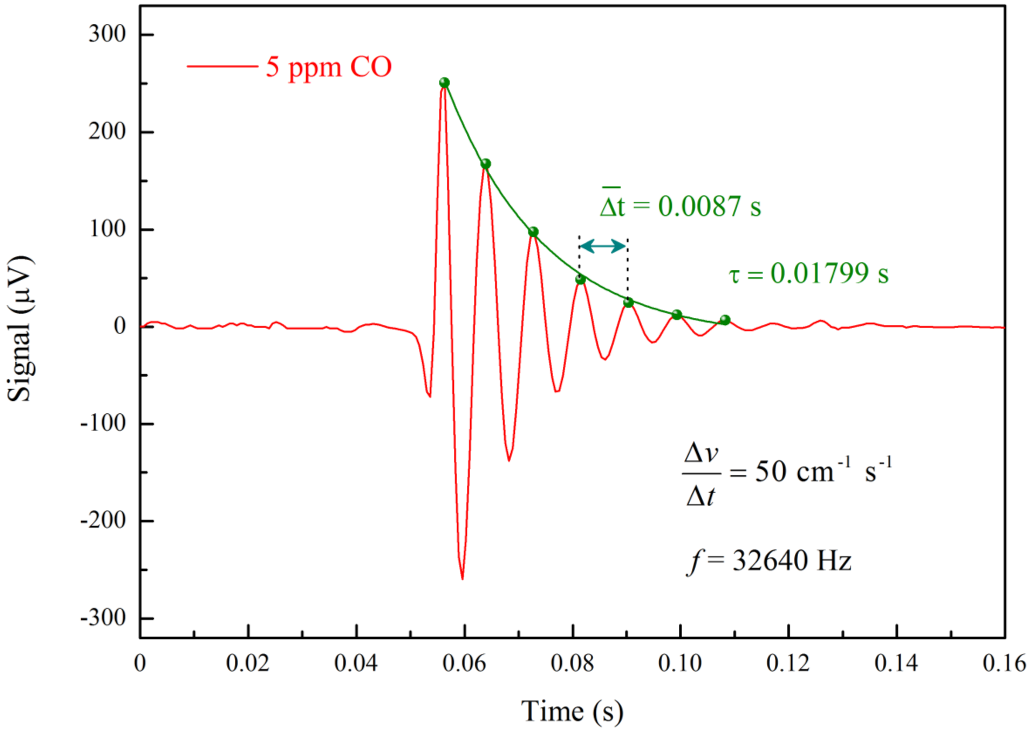

Supplementary Figure 6: Measured BF-QEPAS spectra of 5 ppm CO using a DFB quantum cascade laser emitting at 4.5 μm. The experiment was carried out with the same system as described in this paper (see Figure. 2) at room temperature and a pressure of 700 Torr. The **DFB quantum cascade laser** (DFB-QCL) (AdTech Optics, Inc. Model HHL-14-32) temperature and current were selected to target a CO absorption line located at 2,190.02 cm^{-1} with a 26.5 mW output power and line-strength of 2.915×10^{-19} cm mol^{-1}. The mid-infrared laser beam was focused by a 50 mm focal length plano-convex CaF_2 lens. The DFB-QCL wavelength was scanned at the rate of 50 cm^{-1} s^{-1} by scanning the drive current. A sinusoidal signal with a frequency of 32,640 Hz was applied in order to modulate the DFB-QCL wavelength. The modulation depth was 0.085 cm^{-1}. The time constant and filter slope of the LIA were set at 3 ms and 12 dB, respectively.

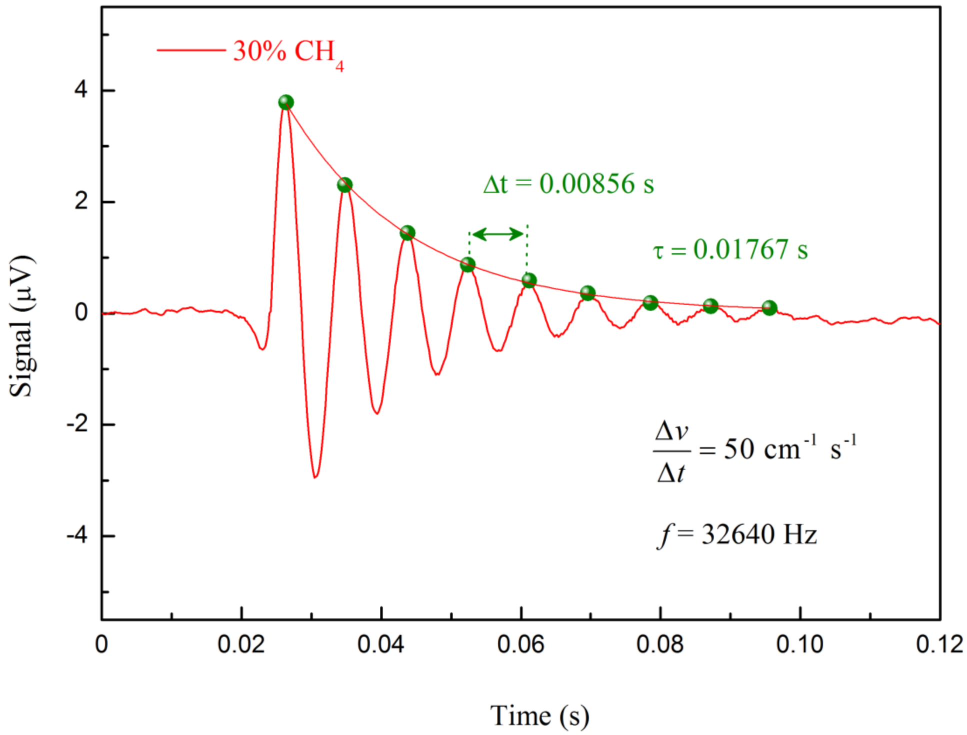

Supplementary Figure 7: Measured BF-QEPAS spectra of 30% CH_4 using a DFB interband cascade laser emitting at 3.6 μm. The experiment was carried out with the same system as described in this paper (see Figure. 2) at room temperature and a pressure of 700 Torr. The **DFB interband cascade laser (**DFB-ICL) (Nanoplus Nanosystems and Technologies GmbH, S/N: 1485/25-19) temperature and current was selected to target a CH_4 absorption line located at 2,778.64 cm^{-1} with a 2.3 mW output power and line-strength of 5.241×10^{-22} cm mol^{-1}. The mid-infrared laser beam was focused by a 30 mm focal length plano-convex CaF_2 lens. The DFB-ICL wavelength was scanned at the rate of 50 cm^{-1} s^{-1} by the scanning the drive current. A sinusoidal signal with a frequency of 32,640 Hz was applied in order to modulate the DFB-ICL wavelength. The modulation depth was 0.497 cm^{-1}. The time constant and filter slope of the LIA were set at 3 ms and 12 dB, respectively.

Supplementary Note 1: Simulation of the responses of the QTF using MATLAB.

Based on Newton's law of motion equation

$$L\frac{\mathrm{d}^2q}{\mathrm{d}t^2}+R\frac{\mathrm{d}q}{\mathrm{d}t}+q\frac{1}{C}=U(t) \quad (1)$$

when a sinusoidal dither is applied to the laser current at a frequency of f, the QTF response can be expressed as:

$$L\frac{q(n+1)-q(n-1)-2q(n)}{h^2}+R\frac{q(n+1)-q(n)}{h}+\frac{1}{C}q(n)=U(t) \quad (2)$$

$$U(t)=A\sin(\frac{1}{f}t)M(t) \quad (3)$$

where h is the step-length and $M(t)$ is the expression of the harmonic component. We define t_a = $h\times n$ as the time of the acoustic force effect with the QTF, which represents the wavelength scanning rate. The value of constant A represents the intensity of the acoustic force. When the parameters of the QTF (L = 5,686 H, R = 633.98 kΩ, C = 4.15×10^{-15} F) are included in the MATLAB program, we can simulate different cases of the QTF resonance by changing the value of A, T_a and f. In addition, the Butterworth filter was used in the MATLAB program to obtain the filter function.

Supplementary Note 2: Optimization of the LIA parameters for the BF-QEPAS sensor

The equivalent noise detection bandwidth (ENBW) of the LIA is determined by its time constant and filter slope, which has a direct influence on the signal-to-noise ratio (SNR) of the system. Unlike traditional QEPAS-based sensors, the ENBW of the LIA in BF-QEPAS based sensor is larger than QTF's ENBW[2]. However, the large detection bandwidth results in a high background

noise. We optimized LIA filter slope and time constant, respectively, in order to obtain a detection bandwidth which does not distort the BE-QEPAS signal and maintains an efficient background noise suppression.

As shown in Supplementary Figure 2, the BF-QEPAS signals increase with a decrease of the LIA filter slope, i.e. the wider LIA ENBW for the same time constant. However, more noise is also detected. For the reported sensor, there was no excessive noise observed when the filter slope decreased from 24 dB to 12 dB. With a further decrease of filter slope, such as 6 dB, the noise increased and became apparent. The LIA time constant was optimized with a similar method. As shown in Supplementary Figure 3, with the same filter slope, the SNR of the BF-QEPAS based sensor increased with the LIA time constant decreasing from 100 ms to 100 μs. However, a further decrease of the time constant led to the sensor to detect more noise, thereby reducing the sensor's SNR. We chose 12 dB and 100 μs as the LIA filter slope and time constant, corresponding to a detection bandwidth of Δf = 1,250 Hz. These experimental results proved that such settings do not distort the line shape of the beat signal and yield the best SNR.

The wavelength scanning rate has a direct influence on the result of the BF-QEPAS based sensor. As described in this paper, the wavelength scanning rate must be fast enough to induce the transient response of the QTF. Based on the vibrational-translational (V–T) relaxation rate of the targeted trace gas, there is an optimal value for this rate. The results plotted in Supplementary Figure 4 show three different cases of the wavelength scanning rates. When the wavelength is scanned at 18 cm^{-1} s^{-1}, the steady state response affects the beat signal and changes the signal shape. When the wavelength scanning rate is too fast, such as 72 cm^{-1} s^{-1}, the signal decreased as the energy absorbed by the gas cannot be effectively transformed to acoustic energy. Hence, the

optimal wavelength scanning rate was experimentally determined to be 36 cm^{-1} s^{-1} for the detection of water vapor.

Supplementary Note 3: Beat frequency signal analysis

As mentioned in the manuscript section entitled "Theory of BF-QEPAS", the beat frequency signal is generated after a pulsed acoustic wave excited the QTF prongs to vibrate in a short period of time. Hence, the first peak/valley of the BF-QEPAS signal, marked by 'O' as shown in Supplementary Figure 5a, is influenced by the acoustic wave. The peak/valley 'O' should not be used for calculating the trace gas concentration. The relationship between the peak/valley values of the beat frequency signal and the gas concentration was investigated. Only the first three peaks A-C/valleys A'-C' of the beat frequency signal, except the peak/valley O, were considered, since the value of the other peaks/valleys are significantly smaller than the first three. As shown in Supplementary Figure 5b, for either peaks or valleys of the BF-QEPAS signal, the linearity of the sensor response to H_2O concentrations was confirmed by the high R-square value (> 0.999) of the linear fitting. Since the larger amplitude contributes to achieving an improved detection limit, a signal analysis was carried out from the first signal peak, except the peak/valley O.

Supplementary Note 4: Performance of BF-QEPAS sensor with three different types of semiconductor lasers

As the BF-QEPAS technique is independent of the excitation wavelength, it was possible to verify the performance of this technique using different kinds of semiconductor lasers. CO and CH_4 were selected as the target gas, respectively. Since CO and CH_4 have a slow V–T relaxation rate, the beat frequency signal is weak when the laser wavelength is scanned rapidly. To obtain a good beat frequency signal, we chose a slower scanning rate and a modulation frequency which

is closer to the resonance frequency compared to the parameters value used in water vapor detection. A 3 ms integration time was used to improve the SNR when the BF-QEPAS signals of the CO and CH_4 were detected. The results shown in Supplementary Figure 6 and Supplementary Figure 7 indicate that the BF-QEPAS technique can obtain accurate values of QTF frequency and *Q* factor. The minimum detection limit and corresponding NNEA coefficient for CO are 10 ppb and 2.3×10^{-8} cm^{-1} W $Hz^{-1/2}$, respectively, while these values are 40.75 ppm and 1.3×10^{-8} cm^{-1} W $Hz^{-1/2}$ for CH_4, respectively.

Supplementary References:

1. Dong, L., Kosterev, A. A., Thomazy, D. & Tittel, F. K. QEPAS spectrophones: design, optimization, and performance. *Appl. Phys. B* **100**, 627-635 (2010).
2. Kosterev, A. A. et al. QEPAS detector for rapid spectral measurements. *Appl. Phys. B* **100**, 173-180 (2010).
